# Stacked Deep Learning Ensemble for Multiomics Cancer Type Classification: Development and Validation Study

**DOI:** 10.2196/70709

**Published:** 2025-08-12

**Authors:** Amani Ameen, Nofe Alganmi, Nada Bajnaid

**Affiliations:** 1 Faculty of Computing and Information Technology, King Abdulaziz University, P.O.Box 80200, Jeddah, 21589, Saudi Arabia, 966 126400000; 2 Institute of Genomic Medicine Sciences (IGMS), King Abdulaziz University, Jeddah, Saudi Arabia

**Keywords:** deep learning, ensemble learning, cancer classification, omics data, stacking ensemble

## Abstract

**Background:**

Cancer is one of the leading causes of disease burden globally, and early and accurate diagnosis is crucial for effective treatment. This study presents a deep learning–based model designed to classify 5 common types of cancer in Saudi Arabia: breast, colorectal, thyroid, non-Hodgkin lymphoma, and corpus uteri.

**Objective:**

This study aimed to evaluate whether integrating RNA sequencing, somatic mutation, and DNA methylation profiles within a stacking deep learning ensemble improves cancer type classification accuracy relative to the current state-of-the-art multiomics models.

**Methods:**

Using a stacking ensemble learning approach, our model integrates 5 well-established methods: support vector machine, k-nearest neighbors, artificial neural network, convolutional neural network, and random forest. The methodology involves 2 main stages: data preprocessing (including normalization and feature extraction) and ensemble stacking classification. We prepared the data before applying the stacking model.

**Results:**

The stacking ensemble model achieved 98% accuracy with multiomics versus 96% using RNA sequencing and methylation individually, 81% using somatic mutation data, suggesting that multiomics data can be used for diagnosis in primary care settings. The models used in ensemble learning are among the most widely used in cancer classification research. Their prevalent use in previous studies underscores their effectiveness and flexibility, enhancing the performance of multiomics data integration.

**Conclusions:**

This study highlights the importance of advanced machine learning techniques in improving cancer detection and prognosis, contributing valuable insights by applying ensemble learning to integrate multiomics data for more effective cancer classification.

## Introduction

Cancer is a complex worldwide health problem associated with high mortality [1]. Recent years have seen the use of a variety of machine learning techniques applied to high-throughput sequencing technology, which has advanced the classification of cancers based on omics data and offered a promising future for precise treatment choices.

Omics data provide a thorough understanding of biological systems, facilitating research into disease pathways, molecular causes, and ecological dynamics. Omics comprises the following fields: metagenomics (eg, microbial genomes), proteomics (eg, protein abundances), metabolomics (eg, small molecule concentrations), epigenomics (eg, DNA methylation patterns), and genomics (eg, DNA sequences and mutations) [2]. RNA sequencing is one type of omics data and is a powerful sequencing-based method that enables researchers to discover, characterize, and quantify RNA transcripts across the entire transcriptome [3]. RNA sequencing can tell us which genes are turned on in the cell, their expression levels, and at what time they are turned on or off [4]. This allows scientists to better understand cell biology and evaluate changes that might indicate disease. These data are characterized as high-throughput and high-dimensional [5]. Methylation, an epigenetic process involving the addition of methyl groups to DNA, plays a vital role in gene expression regulation [6]. Aberrant methylation patterns are pervasive in human cancers, impacting carcinogenesis stages and serving as potential biomarkers for cancer diagnosis and prognosis [7,8]. A somatic mutation is a permanent change that can arise naturally or be brought about by environmental influences in the DNA sequence of a gene or chromosome. It may have an impact on the structure or function of proteins. In cancer research, they are essential markers that shed light on the genetic causes of carcinogenesis and inform the creation of patient-specific targeted therapy [9].

Studies have shown that while single-genome research has yielded significant results, integrating multiple omics can enhance our understanding of diseases and provide patients with better treatment options. Therefore, integrating data from multiple omics, rather than using single-omic techniques, may provide a better understanding of biological systems and the causes of diseases. This integration improves prediction accuracy and facilitates more efficient identification of therapeutic targets [10,11].

Dealing with omics data poses several challenges, one of which is that sequencing data are high-dimensional. Second, class imbalance in patient data will reduce the model’s performance. The third challenge is that the number of patients in the study is still relatively small, which may cause overfitting problems [12]. Based on these challenges, there is a need for development and contribution in this field, including the development of models that can successfully distinguish between types of cancer while considering the 3 challenges.

Recent studies on the analysis of critical data for cancer disorders have used a variety of machine learning strategies, including the multilayer perceptron [13-16]. The multilayer perceptron is a 3-layer system that consists of an input layer, an output layer, and a hidden layer positioned in the middle. A convolutional neural network (CNN) [17,18] is another kind of neural network that is used. It functions similarly to a feed-forward neural network and consists of a convolutional layer that processes the input and outputs the result to the next layer. They also used random forest (RF) [13,19], which is a technique that involves training a large number of decision trees. The final output of the RF is the class that the majority of the trees select. Deep neural architectures for classification have also been used in [18,20,21]. In addition, the support vector machine (SVM) and k-nearest neighbors (KNN), which are typically used for regression and classification, are commonly applied in this field.

Working with omics data presents several challenges, such as overfitting and class imbalance, which we outline below, along with an overview of how previous work has addressed them. Overfitting is common due to the limited amount of data, often resulting in lower model performance. The model’s accuracy is directly influenced by the amount of data used. This issue has been noted in several studies where models are excessively trained to fit the training examples. Upon review, some papers overlooked this issue, while others addressed it through approaches such as regularization, cross-validation, and dropout techniques. Class imbalance is another significant issue in this type of data, affecting model training by biasing it toward the class with more data. Summarizing the methods for dealing with this problem involves 2 main approaches. First, oversampling techniques such as SMOTE (Synthetic Minority Oversampling Technique) and undersampling methods such as downsampling are used to balance class distribution in the dataset. Second, another effective strategy is to use ensemble learning, where different models are trained on either different subsets of data or using various algorithms, pooling their predictions for improved overall performance. These methods collectively aim to address the challenges posed by class imbalance in data-driven tasks such as cancer classification using omics data.

The model proposed in this paper uses ensemble learning of 5 common models to classify the 5 most common types of cancer in the Kingdom of Saudi Arabia using 3 types of omics data. The objective is to investigate whether the model’s classification accuracy improves upon integrating multiomics data into our stacking model, which combines 5 of the most popular methods in this field.

## Methods

### Overview

**Figure 1.**
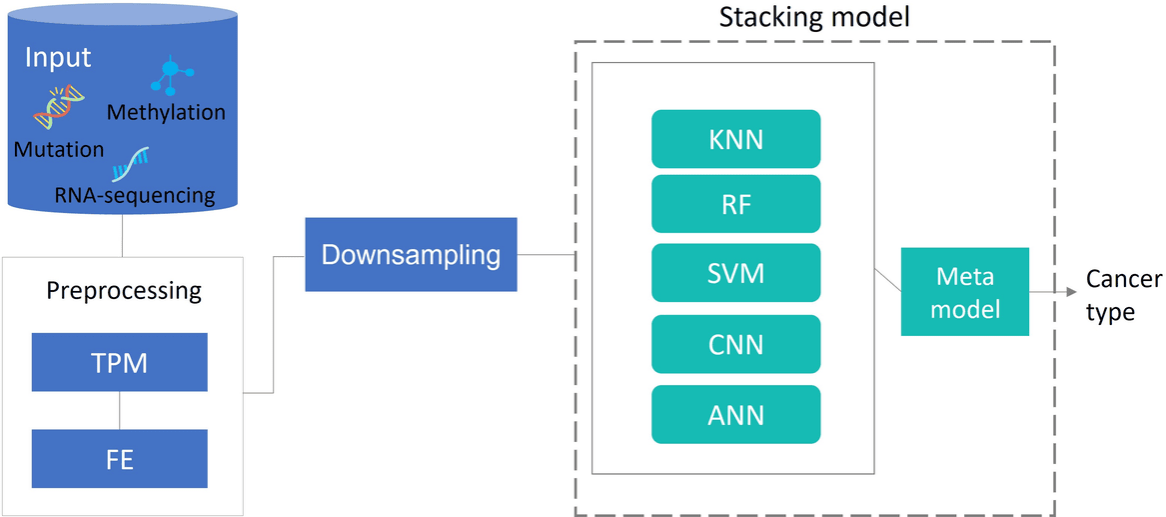
Methodology of the proposed model. ANN: artificial neural network; CNN: convolutional neural network; FE: feature extraction; KNN: k-nearest neighbors; RF: random forest; SVM: support vector machine; TPM: transcripts per million.

Our proposed model presents a classification of the 5 most common types in the Kingdom of Saudi Arabia, which are breast, colorectal, thyroid, non-Hodgkin lymphoma, and corpus uteri [22], by using deep learning, which in turn extracts features that are believed to have an important role. The model was designed using stacking ensemble learning as shown in Figure 1, which goes through 2 phases: a preprocessing phase that includes normalization and feature extraction (FE), and a classification phase using an ensemble stacking model. Data entered the preprocessing phase, and the output was then directed to the stacking model. We have performed our experiments in Python 3.10 (Python Software Foundation) on the Aziz Supercomputer of King Abdulaziz University, which is the second fastest supercomputer in the Middle East and North Africa region. The following sections explain how the proposed model works, starting with data collection, followed by preprocessing, and ending with the stacking model.

### Data Collection and Preprocessing

For RNA sequencing data in this investigation, we used The Cancer Genome Atlas (TCGA) dataset, which is openly accessible to researchers. TCGA comprises approximately 20,000 primary cancer and matched normal samples across 33 cancer types, including the 5 cancer types addressed in our work. Its main goal is to provide scientists with information to improve cancer detection, treatment, and prevention [23]. Furthermore, somatic mutation and methylation data were obtained from the publicly accessible LinkedOmics dataset, which includes multiomics data from all 32 TCGA cancer types and 10 Clinical Proteomic Tumor Analysis Consortium (CPTAC) cohorts [24].

**Figure 2.**
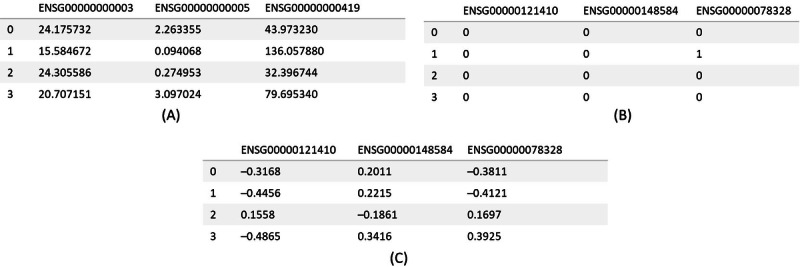
Show screenshots of the data types: (A) RNA sequencing, (B) somatic mutations, and (C) methylation.

Figure 2 shows a screenshot of the data types. These are tabular data, with columns representing genes and rows representing cases that are infected by cancer. In Figure 2A, RNA sequencing data capture gene expression levels as continuous values. In Figure 2B, somatic mutation data are sparse and binary (0 or 1), indicating the presence of genomic alterations. In Figure 2C, methylation data provide continuous epigenetic information reflecting gene regulation patterns, with values ranging from −1 to 1.

Initially, the data underwent extensive cleaning to ensure the integrity of the model by identifying and removing 7% of cases with missing or duplicate values. Table 1 describes the number of cases of the 5 types of cancer after preprocessing. Regarding RNA sequencing data, preparation is required before use to provide a precise model evaluation. Thus, 2 processes were carried out in order to preprocess the data: normalization and Feature Extraction (FE).

**Table 1.** Show the number of samples in each cancer type after preprocessing.

Cancer type	Abbreviation	RNA sequencing	Somatic mutation	Methylation
Breast	BRCA^a^	1223	976	784
Colorectal	COAD^b^	521	490	394
Thyroid	THCA^c^	568	496	504
Non-Hodgkin lymphoma	NHL^d^	481	240	288
Corpus uteri	UCEC^e^	587	249	432

a BRCA: breast invasive carcinoma.

b COAD: colon adenocarcinoma.

c THCA: thyroid carcinoma.

d NHL: non-Hodgkin lymphoma.

e UCEC: uterine corpus endometrial carcinoma.

Next, for the normalization step, we used the transcripts per million method to eliminate systematic experimental bias and technical variation while maintaining biodiversity. In addition, it can reduce the bias resulting from the choice of technique used and the conditions tested, or from the experimental procedure, and it can reduce the variance resulting from natural variation and measurement precision [25]. Transcripts per million can be calculated by equation 1 and should be read as “for every 1,000,000 RNA molecules in the RNA-seq sample, x came from this gene/transcript” [26].


(1) $$\text{TPM}=10^{6}\times \frac{reads\;mapped\;to\;transcript\;/\;transcript\;length}{sum\;(reads\;mapped\;to\;transcript\;/\;transcript\;length)}$$

### Feature Extraction

RNA sequencing data are high-dimensional. Therefore, to reduce the dimensionality, we use an autoencoder technique based on the results of a study [27] that concluded that autoencoders perform effectively while preserving essential biological properties, allowing for better visualization and interpretation of complex data structures. The architecture of the autoencoder model is composed of an encoder, a code, and a decoder. The encoder compresses the input (features), and the decoder attempts to recreate the input (features) from the compressed version provided by the encoder. The autoencoder model has 5 dense layers, each with 500 nodes and a rectified linear unit (ReLU) activation function. A dropout of 0.3 was applied to handle the overfitting.

### Methods for Handling Class Imbalances

**Figure 3.**
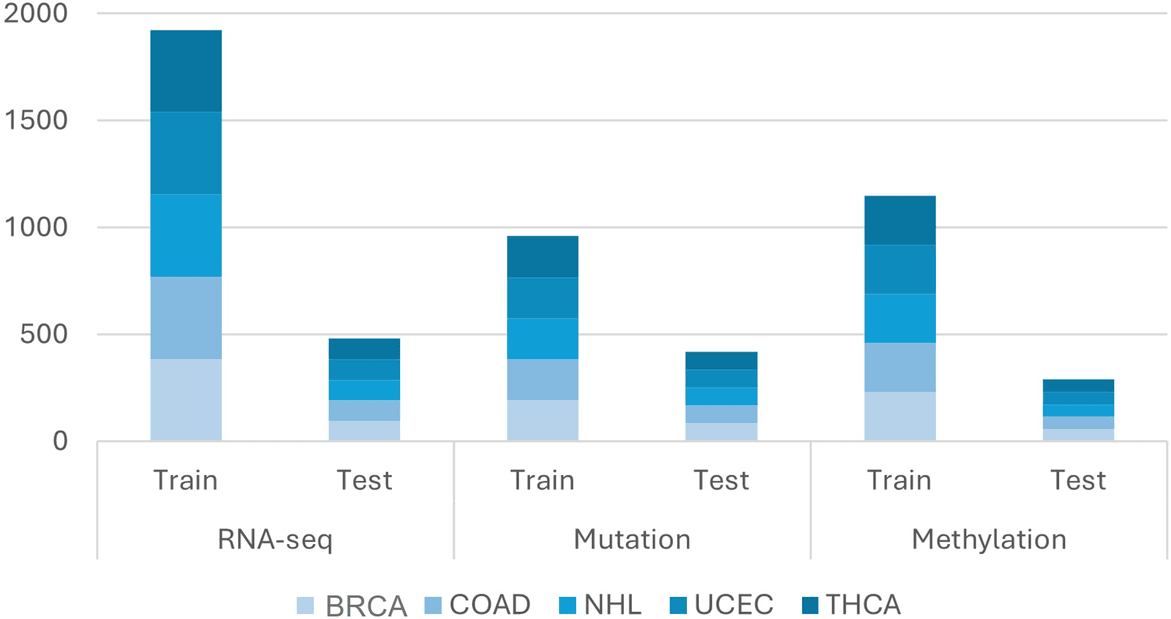
Downsampling for data. NHL had 481 cases in RNA-seq data; 80% (385 cases) were allocated for training and 20% (96 cases) for testing. Somatic mutation types were downsampled to 80% (192 cases) for training and 20% (48 cases) for testing. Methylation data followed suit, with 80% (230 cases) and 20% (58 cases) for training and testing, respectively. BRCA: breast invasive carcinoma; COAD: colon adenocarcinoma; NHL: non-Hodgkin lymphoma; RNA-seq: RNA sequencing; THCA: thyroid carcinoma; UCEC: uterine corpus endometrial carcinoma.

In particular, for classes with tiny sample sizes, imbalanced class sizes in the dataset may result in subpar prediction accuracy. Downsampling and SMOTE are 2 methods used to address class imbalances and enhance model performance [28]. In the study by Dittman et al [29], researchers tried class oversampling and class undersampling; then, after evaluating the data, they concluded that undersampling has better results than the oversampling method. Therefore, we decided to apply the downsampling method for the data used in this paper and verified that the data were free of duplicates and then divided into 80% training and 20% test data (Figure 3).

In this dataset, the smallest class (ie, non-Hodgkin lymphoma) included 481 cases in the RNA sequencing data. To balance the dataset, 481 cases were randomly selected from each of the other classes. This resulted in 80% (385 cases) used for training and 20% (96 cases) for testing. For somatic mutations data, each of the 5 types was downsampled to 80% (192 cases) for training and 20% (48 cases) for testing. Similarly, for methylation data, 80% (230 cases) were assigned for training and 20% (58 cases) for testing.

### Stacking Ensemble Model

Stacking builds a model with improved performance by training multiple models to come up with the best combination of predictions from these models. The model structure consists of 5 base models and a meta-model that collects the predictions of the base models.

The hyperparameters of each model were described using GridSearchCV (scikit-learn developers), providing a comprehensive configuration for testing and optimization. For the nearest neighbor classifier (BM1), GridSearchCV was used to discover the optimal number of neighbors from values of (1, 3, 5, 10, 5, and 0) and found that the optimal number of neighbors was 10. For the RF classifier (BM2), GridSearchCV was used to explore combinations of “n_estimators” and “min_samples_leaf,” achieving the best performance using 500 trees and a minimum of 2 samples per leaf. For the support vector classifier (BM3), the regularization parameter “C” was tuned across a range of values (0.1, 1, 5, 7, and 10), with C=10 achieving the highest accuracy. For CNN (BM4) and artificial neural network (ANN; BM5), GridSearchCV was used to find the optimal activation function from ReLU and softmax, choose dropout rates from 0.1 to 0.6, and finally find the filter value in CNN. Table 2 shows the hyperparameters that we used in each model. Next, the stacking ensemble uses an ANN as the meta-model to combine predictions from BM1 to BM5. The meta-model architecture consists of a neural network with multiple layers. The first dense layer has 32 units and uses a ReLU activation function, followed by a dropout layer with a 50% rate to reduce overfitting. The second dense layer has 16 units and a ReLU activation function, followed by a dropout layer with a 50% rate. The model ends with an output layer that has 5 units and a softmax activation function, suitable for multiclass classification. The model is trained using an Adam optimizer with a learning rate of 0.001 and sparse categorical cross-entropy loss. The integration of the 5 models (SVM, KNN, ANN, CNN, and RF) follows a stacking ensemble approach, where the predictions from each model serve as input features for the meta-model. These base models are trained independently, and their outputs are concatenated to form the input layer of the meta-model.

**Table 2.** Hyperparameters of each base model.

Model	Classifier	Hyperparameter
BM1	KNN^a^	Neighbors=10
BM2	RF^b^	n_estimators=500 and min_samples_leaf=2
BM3	SVM^c^	C=10
BM4	CNN^d^	Conv1D with filters= 64, activation=“ReLU,” optimizer= “adam,” loss= “sparse_categorical_crossentropy,” and dropout=0.3
BM5	ANN^f^	3 dense layers, activation=”ReLU,” “softmax,” optimizer=“adam,” loss=“sparse categorical crossentropy,” and dropout=0.4

a KNN: k-nearest neighbor.

b RF: random forest.

c SVM: support vector machine.

d CNN: convolutional neural network.

e ReLU: rectified linear unit.

f ANN: artificial neural network.

### Ethical Considerations

This study exclusively used publicly available datasets obtained from TCGA and LinkedOmics with project names “TCGA-BRCA,” “TCGA-COAD,” “TCGA-THCA,” “TCGA-DLBC,” and “TCGA-UCEC”. All datasets were fully anonymized and complied with the respective repository’s data usage policies.

## Results

### Overview

In this section, we present the results of our study. First, in the “Performance Evaluation Metrics” section, we analyze critical metrics including the classification report, the confusion matrix, and the receiver operating characteristic (ROC) curve. Second, we present the results of the 5 models individually to compare with our results.

### Performance Evaluation Metrics

**Figure 4.**
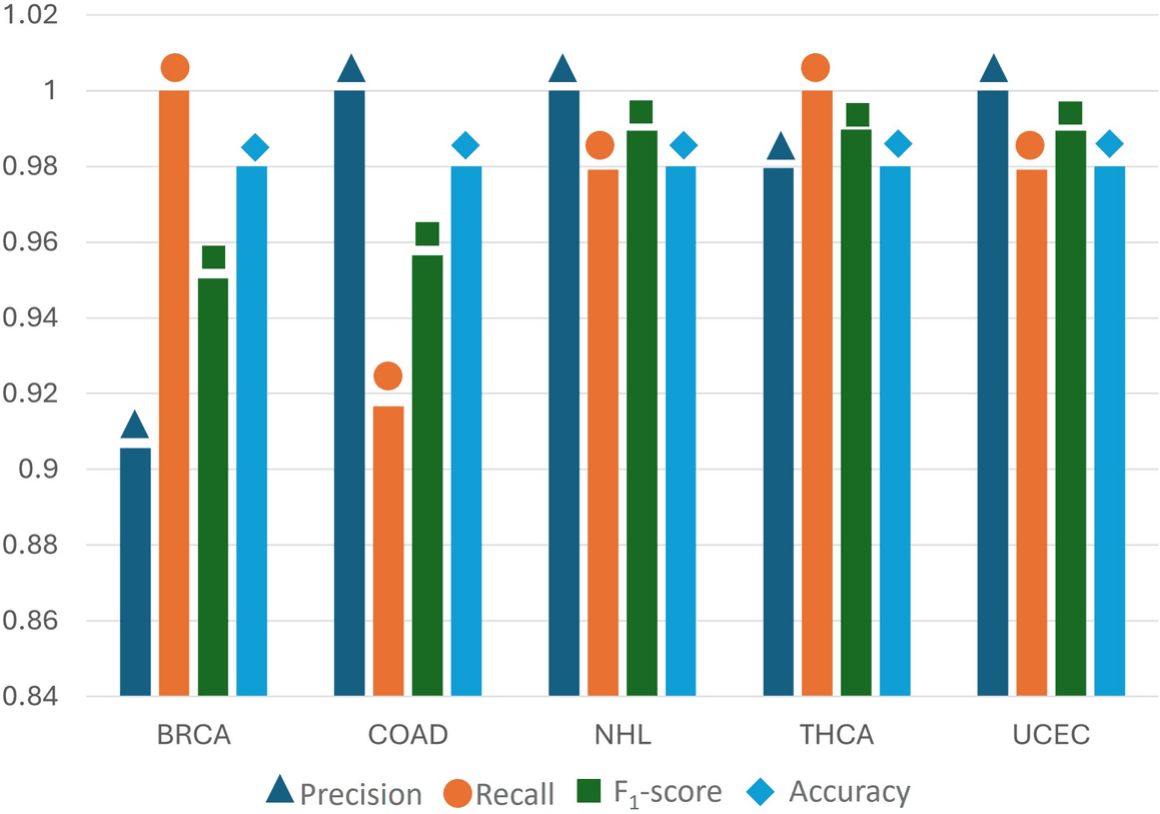
Classification report visualizing precision, recall, *F*_1_-score, and support for each class in the stacking ensemble model. BRCA: breast invasive carcinoma; COAD: colon adenocarcinoma; NHL: non-Hodgkin lymphoma; THCA: thyroid carcinoma; UCEC: uterine corpus endometrial carcinoma.

**Figure 5.**
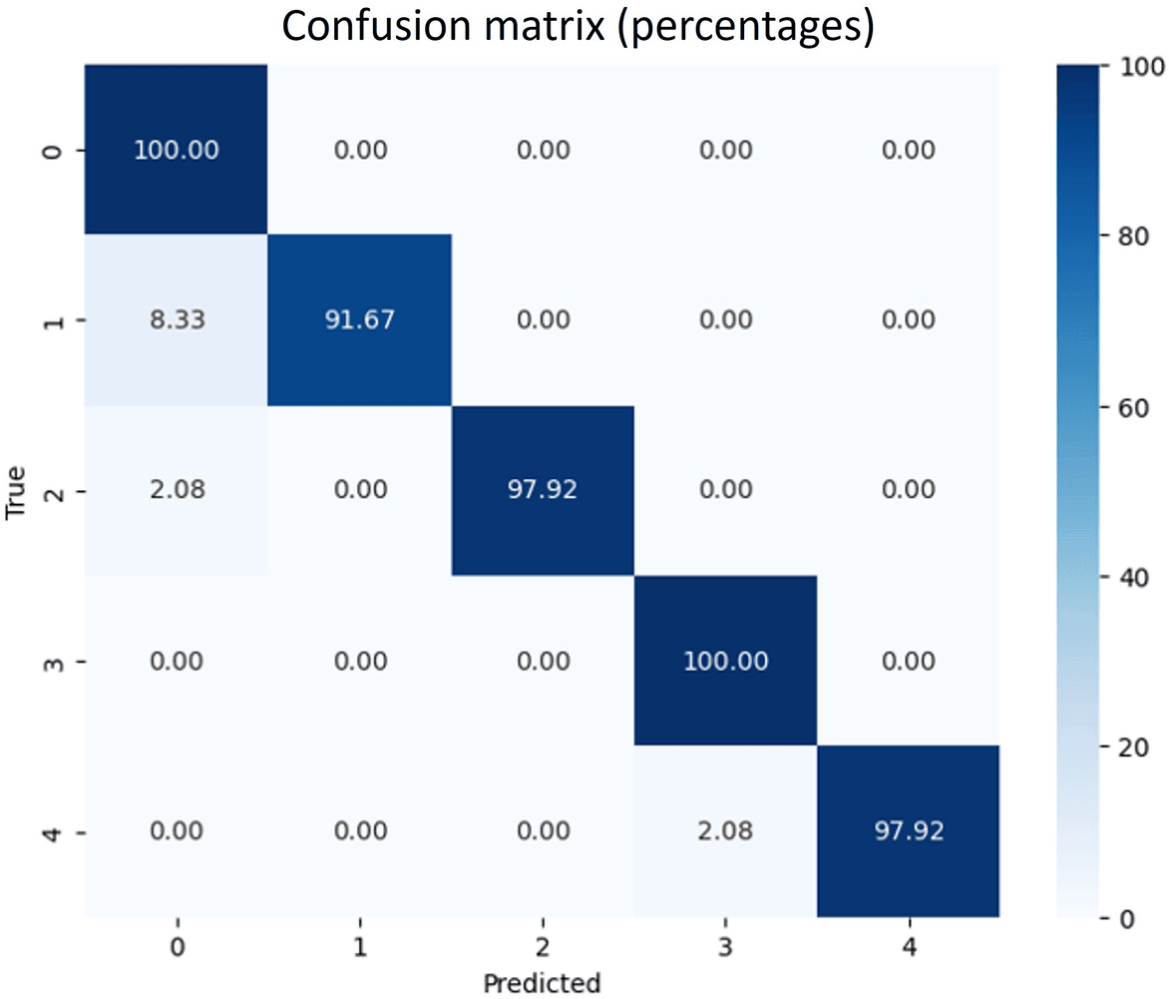
Confusion matrix illustrating the true versus predicted classifications generated by the stacking ensemble model.

To assess the effectiveness of the multiclass classification model, various performance metrics were calculated and are shown in Figure 4. The graph shows the performance metrics for a multiclass classification model, including precision, recall, and *F*_1_-score for each class. Precision indicates the accuracy of positive predictions, while recall measures how many actual positives were correctly identified. The *F*_1_-score balances precision and recall. The model achieved an overall accuracy of 98%. Both the macro and weighted averages of the metrics are very similar, reflecting consistent performance across all classes. Subsequently, in Figure 5, we examined the confusion matrix to assess the model’s classification performance across the 5 classes. The matrix percentages indicated that the correct classification rates (the diagonal values) were between 91.67% and 100%, showing accurate classification results with error rates (the off-diagonal values) of roughly 8% or less for each class.

**Figure 6.**
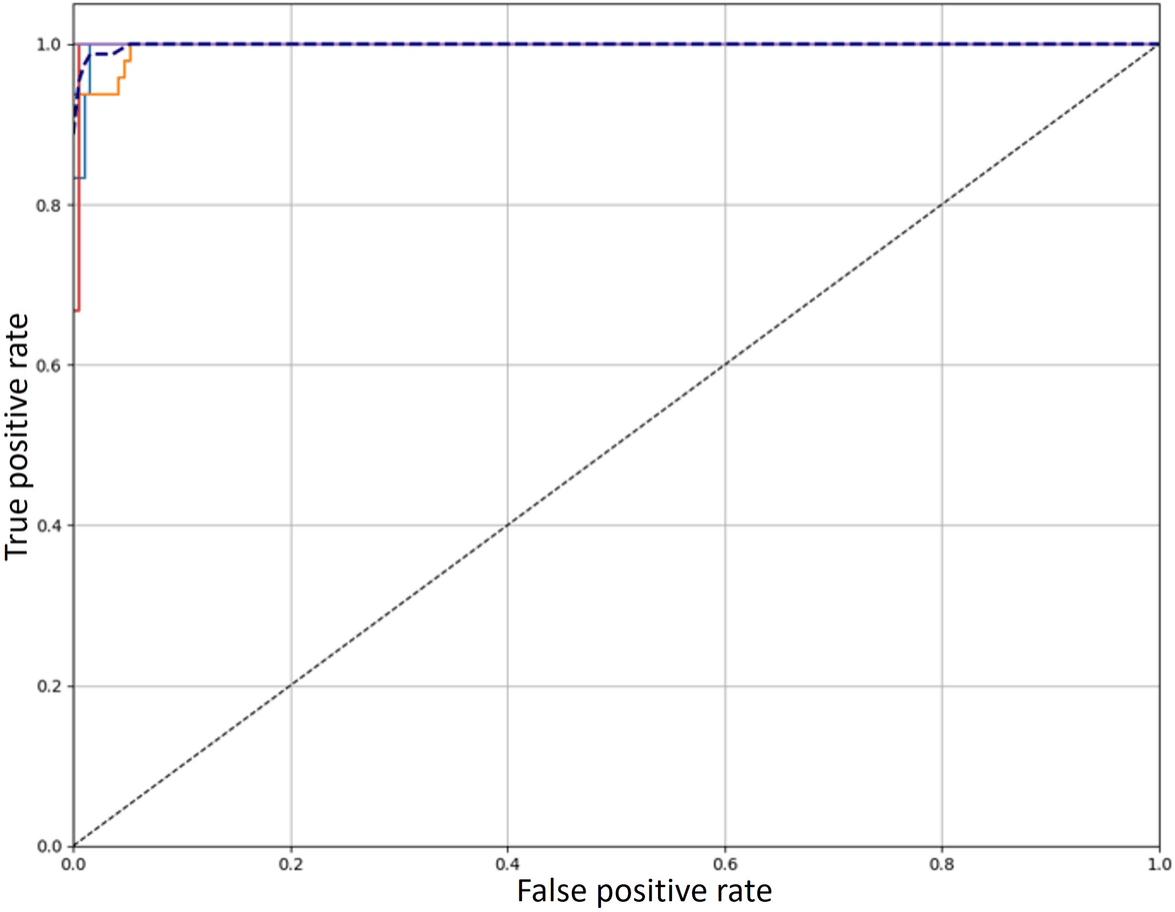
Receiver operating characteristic curve demonstrating the performance of the stacking ensemble model.

Furthermore, we analyzed the ROC curve, which is a tool for assessing the model’s discriminative abilities across multiple classifications. The ROC curve, which provides information about model performance, was modified for our multiclass scenario even though it is usually used in binary classification. In our experiment, we observed compromises between true and false positive rates, which validates the discriminative power of the model. The results, shown in Figure 6, indicate that all classes had consistent performance, as indicated by the area under the curve ranging from 0.90 to 1. These results demonstrate how well the model can classify cases in various classes.

To evaluate the performance of different machine learning approaches on individual omics datasets, we evaluated 5 commonly used classifiers—KNN, RF, SVM, CNN, and ANN—as well as a stacking model composed of all 5 models for each omics type. As shown in Table 3, the RF achieved the highest accuracy on the RNA sequencing dataset (0.98), while the CNN outperformed all other models on the somatic mutations dataset with an accuracy of 0.87. On the methylation dataset, the ANN slightly outperformed the other models with an accuracy of 0.97. The proposed stacking model demonstrated balanced performance across all 3 genome types, achieving accuracies of 0.96 (RNA sequencing), 0.81 (somatic mutations), and 0.96 (methylation). To detail the stacking results, we present Table 4, which shows the performance metrics—precision, *F*_1_-score, recall, and accuracy—for different inputs: RNA sequencing, somatic mutations, methylation separately, and the multiomics approach. For the RNA sequencing input, the model consistently performs well across all 3 folds, with an average precision, *F*_1_-score, recall, and accuracy of 0.96. For the somatic mutations data, the model’s accuracy, *F*_1_-score, and recall were relatively low at 0.60, with a slightly higher precision of 0.70. With a mean of 0.97, the accuracy of the model tested on the methylation dataset varied between 0.95 and 0.99 across folds. Similarly, *F*_1_-score and recall averaged 0.96 and 0.97, respectively, while accuracy averaged 0.96. In the multiomics approach, the model achieved an average score of 0.98 across all metrics. Specifically, the model demonstrates near-perfect performance in folds 2 and 3, achieving a precision, recall, and *F*_1_-score of 0.99, reflecting the added value of incorporating multiple data modalities. Overall, the multiomics approach outperforms using each omics type separately, offering a more robust and accurate model across all evaluation metrics. Our analysis showed that some models performed better in terms of recall and precision for certain cancer types when using multiomics, highlighting the importance of combining data to get the most out of the analysis.

**Table 3.** Classification accuracy of individual models and the stacking model across RNA sequencing, somatic mutations, and methylation datasets.

Classification model	RNA sequencing	Somatic mutation	Methylation
K-nearest neighbors	0.91	0.72	0.95
Random forest	0.98	0.73	0.96
Support vector machine	0.95	0.79	0.96
Convolutional neural network	0.96	0.87	0.96
Artificial neural network	0.96	0.80	0.97
Stacking with the five model	0.96	0.81	0.96

**Table 4.** Performance of the stacking model using RNA sequencing, somatic mutations, methylation, and multiomics data.

Input type and k-fold	Precision	*F*_1_-score	Recall	Accuracy
RNA sequencing				
1	0.95	0.94	0.94	0.94
2	0.97	0.96	0.96	0.96
3	0.98	0.98	0.98	0.98
Avg^a^	0.96	0.96	0.96	0.96
Somatic mutations				
1	0.6	0.6	0.6	0.7
2	0.86	0.85	0.86	0.86
3	0.92	0.91	0.91	0.91
Avg	0.79	0.79	0.79	0.81
Methylation				
1	0.95	0.94	0.94	0.94
2	0.97	0.96	0.97	0.96
3	0.99	0.98	0.99	0.99
Avg	0.97	0.96	0.97	0.96
Multiomics (RNA sequencing, somatic mutations, and methylation)				
1	0.96	0.95	0.95	0.95
2	0.99	0.99	0.99	0.99
3	0.99	0.99	0.99	0.99
Avg	0.98	0.98	0.98	0.98

a Avg: average.

## Discussion

### Principal Findings

The results of this study provide insights into ensemble learning for cancer classification and diagnosis, using 5 different machine learning models. These models were selected based on their proven effectiveness in previous studies and their popularity in the literature, offering a balanced approach to handling the complex nature of multiomics data.

### Comparison With Prior Work

Table 5 summarizes several studies that used multiomics data and machine learning techniques to classify and predict various types of cancer. It is worth noting that these studies are not based on the same data but have been reviewed to support our findings that using multiomics data enhance accuracy. As seen, models from recent studies such as Koh et al [30] and Mohamed and Ezugwu [31] show high area under the curve scores (0.96) and accuracy (97%). Other models, such as Cappelli et al [32] and Jagadeeswara Rao and Sivaprasad [33], also report strong results, typically in the range of 91%‐95%. Overall, these studies highlight the power of integrating multiomics data with advanced machine learning techniques, which consistently led to high accuracy, with models achieving between 91% and 98% accuracy across different cancer types [34]. Although, when comparing the performance of our model with theirs, our approach shows the highest overall accuracy (98%) across a range of cancer types and data modalities. We addressed common challenges in omics data analysis, such as overfitting, class imbalance, and high dimensionality, through the application of techniques such as dropout, downsampling, and FE. These methods significantly contributed to the robustness of our models, though their effectiveness varied depending on the model and data type.

**Table 5.** Comparison of cancer classification performance across multiomics research.

Paper	Year	Data type	Cancer types	Classification model	Overfitting handling	Class imbalance handling	Results (accuracy)
Cappelli et al [32]	2018	RNA sequencing and methylation	BRCA^a^, THCA^b^, and KIRP^c^	C4.5, RF^d^, RIPPER^e^, and CAMUR^f^	Feature regularization methods	N/A^g^	95%
Kwon et al [34]	2023	cfDNA^h^ and CNVs^i^	LUAD^j^	AdaBoost, MLP^k^, and LR^l^	Cross-validation	N/A	91%-98%
Koh et al [30]	2024	Proteomics, RNA sequencing, metabolomics, and targeted immunoassays	Lung	Machine learning	Regularization and QC^m^	Balanced datasets	AUC^n^ 0.96
Jagadeeswara Rao and Sivaprasad [33]	2024	RNA sequencing and methylation	PAAD^o^	Ensemble learning	Ensemble techniques	SMOTE^p^	95%
Mohamed and Ezugwu [31]	2024	RNA sequencing, miRNA^q^, and DNA methylation	LUAD	CNN^r^	Dropout	SMOTE	97%
Our model	2024	RNA sequencing, methylation, and somatic mutations	BRCA , THCA, NHL^s^, UCEC^t^, and COAD^u^	Ensemble learning	Cross-validation and dropout	Downsampling	98%

a BRCA: breast carcinoma.

b THCA: thyroid carcinoma.

c KIRP: kidney renal papillary cell carcinoma.

d RF: random forest.

e RIPPER: Repeated Incremental Pruning to Produce Error Reduction.

f CAMUR: Computer Assisted Molecular Unified Receptor.

g N/A: not available.

h cfDNA: cell-free DNA.

i CNV: copy number variation.

j LUAD: lung adenocarcinoma.

k MLP: multilayer perceptron.

l LR: logistic regression.

m QC: quality control.

n AUC: area under the curve.

o PAAD: pancreatic adenocarcinoma.

p SMOTE: Synthetic Minority Oversampling Technique.

q miRNA: microRNA.

r CNN: convolutional neural network.

s NHL: non-Hodgkin lymphoma.

t UCEC: uterine corpus endometrial carcinoma.

u COAD: colon adenocarcinoma.

Typically, deep learning components benefit from graphics processing unit acceleration and need a large amount of computational power, particularly when trained on high-dimensional clinical data. Nevertheless, after training, the model inference time is rather short, allowing for quick predictions that can assist with clinical decisions made in real time. Even while low-resource systems might not be able to support model training, these pretrained models could be used for clinical deployment, particularly in settings with recent computer technology.

### Strengths and Limitations

Typically, deep learning components benefit from graphics processing unit acceleration and need a large amount of computational power, particularly when trained on high-dimensional clinical data. Nevertheless, the model inference time is rather short after the ensemble has been trained, allowing for quick predictions that can assist with clinical decisions made in real time. Even while low-resource systems might not be able to support model training, pretrained models can be used for clinical deployment, particularly in settings with recent computer technology.

However, the study has several limitations that must be acknowledged. Data availability constraints limited the scope of our analysis, and the absence of clinical data meant that our findings are based solely on omics data. This restricts the generalizability of our results to real-world clinical settings, where the integration of clinical and omics data is crucial for accurate cancer diagnosis and prognosis. Furthermore, the common limitation in omics data is dataset size, which may result in overfitting. Another restriction is the absence of external validation.

### Future Directions

Future research should focus on expanding the types of data used in cancer classification, particularly by incorporating patient clinical data and exploring additional omics layers such as metabolomics and proteomics. Furthermore, the integration of multiomics data with advanced machine learning methods holds promise for deepening our understanding of the molecular mechanisms underlying cancer development. This could lead to more precise cancer staging and prognosis, ultimately improving patient outcomes.

### Conclusions

In conclusion, while our study advances the accuracy of cancer classification algorithms, it underscores the need for continuous improvement and validation in diverse and clinically relevant datasets. By addressing these challenges, future research can enhance the applicability of these models in clinical practice, contributing to more effective cancer detection and treatment strategies.

The study aimed to investigate whether incorporating multiomics data into a stacking model that integrates 5 key methods, namely SVM, KNN, ANN, CNN, and RF, enhances the model’s ability to classify cancer. With multiomics, the stacking ensemble model obtained 98% accuracy, compared to 96% with RNA sequencing and methylation separately and 81% with somatic mutation data. It emphasizes the importance of integrating advanced machine learning techniques into health care for more effective cancer detection and prognosis. This highlights the need for continuous improvement and validation of classification models in real-world clinical settings to maximize their impact on cancer care. Future research should focus on incorporating clinical metadata and multiomics data to enhance cancer classification, which would improve patient outcomes and clinical applicability.

## Abbreviations

ANN: artificial neural network
CNN: convolutional neural network
FE: feature extraction
KNN: k-nearest neighbors
ReLU: rectified linear unit
RF: random forest
ROC: receiver operating characteristic
SMOTE: Synthetic Minority Oversampling Technique
SVM: support vector machine
TCGA: The Cancer Genome Atlas
